# Virulence Factors Variation Among *Bordetella Pertussis* Isolates in Iran

**Published:** 2015

**Authors:** Vajiheh Sadat Nikbin, Nazanin Jannesar Ahmadi, Maryam Hosseinpour, Masoumeh Nakhost Lotfi, Fahimeh Shooraj, Fatemeh Sadeghpour, Fereshteh Shahcheraghi

**Affiliations:** Pertussis Reference Laboratory, Department of Bacteriology, Pasteur Institute of Iran.

Sir,

Pertussis or whooping cough, caused by *Bordetella pertussis,* is one of the important vaccine preventable diseases. Pertussis vaccination is recommended as a part of routine childhood immunization. The whole cell pertussis vaccine (WCV) combined with tetanus and diphtheria toxoids (DTP) was introduced in the 1950s in many countries (1-3).

A new form of vaccine called "Acellular vaccine (ACV)" that contained a limited number of defined proteins, was introduced in many developed countries using this vaccine vaccinating their populations for nearly 14 years. This vaccine replaced WCV to avoid the side effects of the latter vaccine in some countries (4). Although the incidence of pertussis was successfully reduced after the introduction of both types of vaccines, the disease is still endemic and the resurgence of pertussis has been revealed in many countries even with high vaccination coverage (5-8). It has been suggested that among the several reasons of this re-emergence (9), genetic diversity in virulence factors is one of the major causes that has affected vaccine efficacy (10-13).

Pertussis toxin (Ptx) and pertactin (Prn) are two virulence factors used in ACV vaccine which confer protective immunity in animals and humans (14).

The gene encoding fimbriae is the other virulence gene which is also subject to variation due to mutation. *B. pertussis* strains contain Fim2 and/or Fim3 fimbriae serotypes. Two and four alleles of *fim2* and *fim3* genes have been identified up to now (15). Packard et al. in 2004 showed that as a variation within *cya*A was extremely rare, it may be suitable for inclusion in ACVs (16). Based on studies of *B. pertussis* strains variation, first from the Netherlands (12), it was postulated that the antigenic divergence between vaccine strains and clinical isolates may have contributed to the resurgence of pertussis (17).

Previous publications demonstrated that non-vaccine types of Ptx, Prn and Fim3 (immune factors) replaced the vaccine types in later years and the isolates collected in the vaccination period appeared to be genotypically different from the vaccine strains in use (18-19).

Despite the 99% coverage of pertussis vaccination in Iran, like other countries we also observed an increase in pertussis rate in our country since 2004. The incidence of pertussis was 98, 125 and 650 cases in 2004, 2005 and 2011 (20-22). The polymorphism of the virulence genes of *B. pertussis* in recent years, the relation of this polymorphism to the resurgence of pertussis and also the lack of any information about the allelic variation between the Iranian isolates have promoted us to analyze the genes encoded virulence factors including *ptx*S1, *prn*, *fim3* and *cyaA* to understand the differences between circulating strains and vaccine strain in Iran. We also aimed to compare our finding to the other investigations in European and American countries.

We studied 31 *B. pertussis* isolates collected in different provinces of Iran from patients between 2008 and 2012. Nasopharyngeal Dacron swab samples were obtained from patients with a clinical diagnosis of pertussis and after the transmission of the specimens in the Pertussis Reference Laboratory in Pasteur Institute of Iran, swabs were primarily cultured on fresh Regan-Lowe medium (provided from Difco Laboratories) containing 10% defibrinated horse blood with and without cephalexin (40 μg/ml) (Sigma Chemical Co., USA).

The age distribution of the patients were as follows: 12 patients were aged 2 months or younger, 13 patients 2-18 months old, 5 patients between 18 months and 10 years of age, and one patient 31 years old.

DNA sequencing of the genes encoding the region 1 of pertactin, S1 subunit of pertussis toxin*,* fimbriae 3 and adenylate cyclase toxin was performed using PCR fragments by using the previously described primers (Table 1). Sequencing was carried out using the ABI capillary system (Macrogen Research, Seoul, Korea). Reference strain *B. pertussis* 134 (provided from Razi Vaccine & Serum Research Institute of Iran) used for pertussis vaccine (WCV) in Iran was also studied.

PCR products of the genes studied in this research were 934, 600, 800 and 500 bp for *ptxS1,* *prn *(region 1),* fim3 *and* cya* genes, respectively. Sequencing results of *prn*, *ptxS1, fim3 *and *cya* genes indicated only one allele of the genes among the isolates including *ptxS1A*, *prn2, fim3B *and* cyaA2*, respectively. The alleles of the studied genes were different from vaccine strain 134 (*ptxS1B*/ *prn1/ fim3A/ cyaA2*). These findings showed similarity between our study and the predominant alleles in the world (15).

Vaccination is the best approach to control the isolates similar to vaccine strains but such an approach cannot prevent the spread of the isolates which are different in their virulence factors and bacteria that produce different antigens compared to vaccines, however, will continue to circulate. Recently many studies worldwide have demonstrated that allelic variation in virulence factors of *B. pertussis* has occurred in populations after the introduction of vaccination (4, 23). It was suggested that these variations might be due to strain adaptation. Strain adaptation in vaccination period remarkably resulted in the single nucleotide mutation named SNP (single nucleotide polymorphism) (23). It seems that the vaccination has acted to select the strains by selective pressure and shifted the competitive balance between strains (19).

In many countries, circulating bacteria has different genotypes based on the sequencing of virulence genes compared to the strains used to produce pertussis vaccine (17-19, 24-26). It is possible that polymorphism in pertactin and pertussis toxin is driven by host immunity because of the fact that polymorphic residues of these factors have been implicated in interaction with the host immune system (19, 27).

The results of previous studies in our country (28 and unpublished data) showed a low antibody titer in the serum of vaccinated children with *B. pertussis* suggested waning immunity in our country too. However, it is necessary to investigate the protection provided by the currently pertussis vaccine used in Iran.

Up to now, thirteen *prn* alleles have been described which make them one of the most polymorphic genes in *B. pertussis* (29). Predominant alleles of *prn* are *prn1,prn2* and *prn3* compared to vaccine strains in the world which harbor *prn1*, *prn7* and *prn10* alleles (15). About *ptxS1* gene, *ptxA1* and *ptxA2* predominate in *B. pertussis* population from 5 variations of the *ptx* gene identified. 2 alleles of* fim2* and 3 *fim3* have been found in which *fim2-2* and *fim3-2* are the new ones. The *fim3-2* alelle replaced the vaccine type *fim3-1* in 2001-2005 (15).

In spite of many studies in the world, information about a strain variation of *B. pertussis* from Asian countries is extremely rare. In this research, we tried to find the predominat variants of virulence proteins among circulating *B. pertussis* population in Iran in comparison with strains used for vaccine production. Unfortunately, there are not any isolates from the prevaccination era in Iran. Therefore, we cannot compare the strains circulating at two periods of time, prevaccination and postvaccination due to the absence of information about the historical isolates in our country.

WCV was introduced about 60 years ago and it is still used for vaccination up to now in our population. Despite the long time of the vaccination and the high coverage of vaccination about 99% in our country (22, 30), we still recovered the *B. pertussis* strains from nasopharyngeal specimens of the patients.

The results of allelic variation of this study are the first report of dominant alleles of the strains circulating in our population. Our results showed that all of the 31 strains (100%) are *ptxS1A/prn2/fim3-2/cyaA2* ones that are similar to many European countries and the US according to table 2 (15). The strains used for vaccine in our country are the same like other countries in the world and our results of alleles of the studied genes were also the same. However, different vaccine composition may affect the *B. pertussis* circulating in that population (31-33).

According to the almost the same allelic pattern of the circulating strains in Asia, Europe and America, it seems that *ptxS1A/prn2 *strains displayed a survival advantage over the other strains against vaccines in populations with good coverage of vaccination.

Although, there is no evidence that a new generation of pertussis vaccines controls the disease better than WCV, it is of major interest to design new vaccines matching the proteins existing in circulating bacteria in the population. It should be considered that such designs require the regular collection of bacteria in the populations.

**Table 1:** Primers for amplification and sequencing of the target genes of *B. pertussis*

**Primer name**	**Sequence 5´-3´**	**gene**	**Reference**
AF	CAATGTCACGGTCCAA	region 1 of *prn*	
AR	GCAAGGTGATCGACAGGG		(18)
S1-F2	CCCCTGCCATGGTGTGATC	*ptxS1*	(18)
S1-R2	AGAGCGTCTTGCGGTCGATC		
Fim3F	CCCCCGGACCTGATATTCTGATG	*fim3*	
Fim3R	GCTGAGCGTGCTGAAGGACAAGAT		(34)
cyaA-2F	ACGACACCCTGGTTGGCGGC	*cyaA*	(16)
cyaA-2R	CCTGGATGGATCATGGCGGA		

**Table 2:** Predominant alleles of *ptx *and *prn* genes in the world in recent years

**Country/year**	**Predominant allele**
The Netherlands/ 2001	*ptxS1A */* prn2,3*
France /2001	*ptxS1A* / *prn2*
Argentina/ 2007	*ptxS1A* / *prn2*
The United States /2000	*ptxS1A* / *prn2*
Finland /1999	*ptxS1A */* prn2,3,4*
The Netherlands /1998	*ptxS1A* / *prn2,3*
Sweden/2003	*ptxS1A */* prn2,3,4*
Australia/1997	*ptxS1A* /*prn1 *
Japan/2010	*ptxS1A /prn2*
Poland/2011	*ptxS1A,/ prn1, 2, 3*
Taiwan/2006	*ptxS1A /prn2*
China/2010	*ptxS1A /prn1*
Iran/2014	*ptxS1A/ prn2*

## Conflict of interests

The authors declared no conflict of interests.
